# CodAn: predictive models for precise identification of coding regions in eukaryotic transcripts

**DOI:** 10.1093/bib/bbaa045

**Published:** 2020-05-27

**Authors:** Pedro G Nachtigall, Andre Y Kashiwabara, Alan M Durham

**Keywords:** mRNA, CDS characterization, UTR characterization, annotation

## Abstract

**Motivation:**

Characterization of the coding sequences (CDSs) is an essential step in transcriptome annotation. Incorrect identification of CDSs can lead to the prediction of non-existent proteins that can eventually compromise knowledge if databases are populated with similar incorrect predictions made in different genomes. Also, the correct identification of CDSs is important for the characterization of the untranslated regions (UTRs), which are known to be important regulators of the mRNA translation process. Considering this, we present CodAn (Coding sequence Annotator), a new approach to predict confident CDS and UTR regions in full or partial transcriptome sequences in eukaryote species.

**Results:**

Our analysis revealed that CodAn performs confident predictions on full-length and partial transcripts with the strand sense of the CDS known or unknown. The comparative analysis showed that CodAn presents better overall performance than other approaches, mainly when considering the correct identification of the full CDS (i.e. correct identification of the start and stop codons). In this sense, CodAn is the best tool to be used in projects involving transcriptomic data.

**Availability:**

CodAn is freely available at https://github.com/pedronachtigall/CodAn.

**Contact:**

aland@usp.br

**Supplementary information:**

Supplementary data are available at *Briefings in Bioinformatics* online.

## 1 INTRODUCTION

The structural annotation of sequences resulting from a transcriptome assembly is an important step to understand the profile of genes expressed in the sample [4]. The coding region of the transcripts (CDS) represents the portion of the transcript that defines the resulting protein that will be produced [20]. Also, the untranslated regions (UTRs) are considered crucial to understanding the genetic regulatory networks involved in specific biological pathways [8, 24, 25]. UTRs are major components of post-transcriptional regulation of gene expression (reviewed by [1]). UTRs are responsible to regulate mRNA stability, export, cellular localization and translation efficiency, which influence directly the final amount of protein (reviewed by [27]). Moreover, the complex pattern of UTR regulation is strongly associated with embryogenesis, cellular diversity and diseases [8, 31, 40]. The correct characterization of the UTR and CDS landscape is, therefore, an essential initial step in correctly identifying the functional protein and the post-transcriptional regulatory elements that can determine the final protein output.

Currently, there are several computational tools to detect the CDSs and UTRs of transcripts. Some of these tools focus on characterizing the CDS [7, 23, 34] and others in characterizing the UTR regions [6, 10, 15, 17, 39]. Additionally, some widely used machine learning approaches were developed to classify transcripts as protein-coding genes or non-coding genes [12, 22, 30, 41], but these methods are only classifiers and do not perform annotation of the coding sequences.

There are basically two strategies for the implementation of CDS predictors: similarity search and *ab initio* predictors. Similarity-based methods [10, 26, 38] rely on the existence of curated proteins and are useful for genes that code for closely related curated proteins but fail to characterize CDS for novel proteins. We can separate *ab initio* prediction methods in two categories: (i) pre-trained methods [23, 29] that generally require curated sequences to estimate specific parameters or the use of the pre-computed parameters of the closely related species available; and (ii) self-training methods, which detect putative long ORFs in the transcripts to train a prediction model specific to that set of sequences [3, 5, 7, 11, 34, 35].

The design of a computational tool that can be easily and automatically applied on any species and to strand-specific, strand-blind or partial sequences is necessary for a wide and confident characterization of CDS and UTR landscape in all novel transcriptome projects. Three previous approaches circumvent this problem with a self-training approach, where predictors first perform an expectation maximization (EM) interactive procedure to train the prediction model using the target data and can be applied to any organism: Prodigal [11], TransDecoder [7] and GeneMarkS-T [34]. Of these, GeneMarkS-T [34] presents a performance closer to a gold standard in stop-codon prediction, with an average of more than $90\%$ of correct predictions of stop codons [34]. However, as we will show below, performance decreases when considering full CDS prediction (i.e. correct start and stop codon identification), strand-blind prediction (where the orientation of the transcript is unknown) or partial sequence prediction, indicating the need for new approaches that can reliably characterize CDS in all sequencing scenarios.

Here we present CodAn, a new transcript characterization software for eukaryotic organisms that dramatically increases the current accuracy boundaries in partial and in strand-blind sequences, increases the accuracy in start codon prediction and matches or surpassing gold-standard accuracy for stop codon prediction in strand-specific sequences. Currently, CodAn has four probabilistic models, each for a specific group of eukaryotes: vertebrates, invertebrates, plants and fungi. We show that with these pre-designed models, CodAn can perform highly confident predictions of the full CDS and UTR regions not only in strand-specific full transcript sequences but also in strand-blind and partial sequences in a rate far higher than other available software.

## 2 METHODS

### 2.1 Algorithm implementation

**Figure 1:**
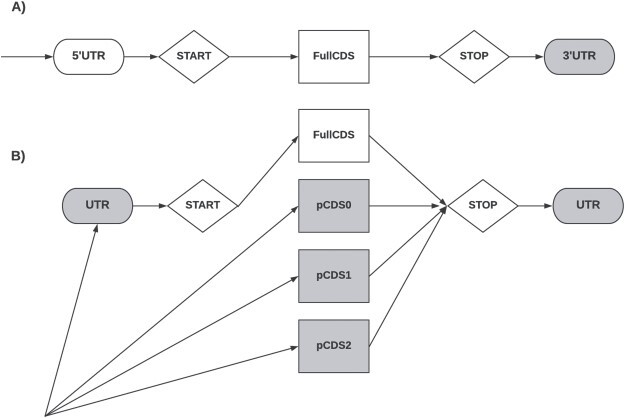
The two GHMMs representing transcripts. (A) Full transcript model, gray figures represent final states, the arrows represent the flow of the architecture, indicating only one initial state, 5$^\prime $UTR. (B) Partial transcript model, gray figures represent final states, the arrows represent the flow of the architecture. The four states represented by circles are states with explicit duration distribution that emit the protein-coding region: fullCDS, pCDS0, pCDS1 and pCDS2. The state fullCDS models a complete coding region. The states pCDS0, pCDS1 and pCDS2 represent partial coding regions that start, respectively, at frame 0, 1 and 2. The state labeled UTR can be used to represent either the 5$^\prime $UTR or the 3$^\prime $UTR. The 3$^\prime $UTR state represents 3$^\prime $UTRs. The states *Start* and *Stop* in diamonds have a fixed length duration, and they represent the start codon and stop codon, respectively.

CodAn uses two different architectures for analyzing transcripts, one for full and one for partial transcripts (Figure 1). The first model applies to transcripts that include the whole CDS region, including the start and stop codon. The partial transcript model assumes that the transcript sequence may not include either the start, or the stop, neither the start or the stop codons. Both architectures assume only one CDS per transcript. These architectures are described using Generalized Hidden Markov Models (GHMMs) implemented using the ToPS probabilistic framework [13]. Since GC content is known to affect gene prediction [33], we partitioned our probabilistic model in GC content specific sub-models. More details on the probabilistic model are described in the SupplementalMethods.

CodAn uses ToPS [13] to implement the GHMM architectures, Python (v.3.6.8) and Perl (v5.26.1) scripts to prepare and process data for the ToPS probabilistic framework.

For each architecture, four different sets of parameters were estimated, corresponding to four organism groups: vertebrates, invertebrates, plants and fungi.

By default, CodAn takes as input transcripts in FASTA format, performs the prediction and returns three FASTA files, containing the CDS, 3$^\prime $UTR and 5$^\prime $UTR sequences predicted for each transcript, and a GTF file, containing the annotations of the predictions for each transcript. CodAn does not need any additional training, the user just specifies the appropriate organism group from the four current choices: vertebrates, invertebrates, plants and fungi. Please contact the authors for customization to new organism groups.

### 2.2 Training sets

CodAn uses probabilistic models for which we need to estimate the parameters. For this, we used training sets with reference sequences from different species downloaded from the RefSeq database at NCBI (release number 94; ftp://ftp.ncbi.nlm.nih.gov/refseq/). Due to a lack of complete annotations of transcripts for *Caenorhabditis elegans* at RefSeq, we used the sequences deposited at the WormBase (release WS270; ftp://ftp.wormbase.org/pub/wormbase/). We retrieved sequences following three criteria: (i) presence of a reviewed and/or curated status; (ii) validated expression status; and (iii) full-length transcripts. We estimated four different parameter sets, each one targeted to a different group of eukaryotic organisms: vertebrates, invertebrates, plants and fungi. The training sets for each parameter set contained reference transcript sequences of a mix of species from each group (detailed in Supplemental Table S1_A). Moreover, we detail the workflow of the training set in the Supplemental_Figure§1.

### 2.3 Comparison protocol

We compared the prediction performance of CodAn against that of ESTscan (v3.0.3; [23]), TransDecoder (v5.5.0; [7]), Prodigal (v2.6.3; [11]) and GeneMarkS-T (v5.1; [34]). We used all tools with default command-line options, following their usage guidelines, as the fine-tuning of the software options of each tool is beyond the scope of this analysis. For ESTscan we used the pre-trained models either of the species being tested or the closest related species when the species-specific model was not available (the pre-trained models used for each species are specified in Supplemental Table S1_B). Since there was no fungi model for ESTScan, we did not perform comparison tests for this tool in the Fungi group. For Prodigal, we used the mode directed to predict intron-less genes (‘switched-off RBS model’), which can be applied to predict coding regions in transcripts of eukaryotes.

We performed a comparison in both full transcript and partial transcript sets. Following Tang and collaborators [34], we used both annotated and Ribo-seq validated full transcripts. The first for evaluating the accuracy of stop codon prediction, the second for evaluating full CDS prediction accuracy. In all tests we measured the Precision (computed as TruePositives / (TruePositives + FalsePositives), Recall (computed as TruePositives / (TruePositives + FalseNegatives)) and F1-score (computed as 2 * (Precision * Recall) / (Precision + Recall)). Following [34], we considered True Positives as the predictions that exactly matched the reference annotation, False Positives as the predictions that presented any difference from the reference annotation and False Negatives as the sequences with no predictions. In this sense, for the annotated full transcripts we considered as True Positives the predictions that correctly matched the annotated stop codon and false positives all other predictions. For both the Ribo-seq validated sequences and for the partial sequences, we considered True Positives all predictions that correctly matched the whole CDS of the transcripts.

We adopted the most common interpretation of the concepts of True Positive, False Positive and False Negatives used in gene prediction. These measures would be sufficient in the ideal situation where all sequences are mRNA transcripts with a CDS region. However, in transcriptome projects sequences with no CDS region can be present, either being just UTRs or, depending on sequencing protocol, ncRNAs. To evaluate the rate of false discoveries, we also compared Specificity (computed as FalsePositives / (FalsePositives + TrueNegatives)) of the various approaches. For this, we used two different negative datasets: 3$^\prime $UTR regions and ncRNAs. It is important to note that here the definition of False Positives is somewhat different from that used in computing Precision, Recall and F1-score: in the latter case false positives were only predictions that did not exactly match the CDS nucleotides, for Specificity we considered any prediction as a false positive.

### 2.4 Testing sets

The test sets for comparison against other approaches consisted of transcript data from 34 eukaryote species that are of interest in the fields of evolutionary and biomedical studies and/or highly used in food production (Table 1). For each of the 34 organisms, we retrieved 2000 randomly selected full transcripts presenting the following three criteria: (i) validated expression status; (ii) full length; and (iii) full CDS annotation. None of these sequences included any of the transcripts used for training the probabilistic model. For each transcript set, we generated seven distinct validation sets: two sets with full transcripts, four sets with partial transcripts and one set with ncRNA sequences (as shown in the flow diagram at the Supplemental_Figure§2).

**Table 1:** Species with validated annotations at RefSeq and used in the present study

**Kingdom**	**Group**	**Species**	**Common Name**
Animals	Vert.	*Anolis carolinensis*	Lizard
		*Bos taurus*	Cow
		*Danio rerio*	Zebrafish
		*Gallus gallus*	Chicken
		*Homo sapiens*	Human
		*Mus musculus*	Mouse
		*Oreochromis niloticus*	Nile tilapia
		*Rattus norvegicus*	Rat
		*Salmo salar*	Salmon
		*Xenopus tropicalis*	Frog
	Inv.	Aedes aegypti	Mosquito
		*Apis mellifera*	Bee
		*Caenorhabditis elegans*	Worm
		*Ciona intestinalis*	Ascidian
		*Drosophila melanogaster*	Fruitfly
		*Nematostella vectensis*	Sea anemone
		*Schistosoma mansoni*	Blood Fluke
		*Tribolium castaneum*	Beetle
Plants	Dico.	*Arabidopsis thaliana*	Arabidopsis
		*Glycine max*	Soybean
		*Olea europaea*	Olive
		*Theobroma cacao*	Cocoa Tree
	Mono.	*Oryza sativa*	Rice
		*Sorghum bicolor*	Sorghum
		*Setaria italica*	Millet
		*Zea mays*	Maize
Fungi		*Agaricus bisporus*	Mushroom
		*Aspergillus niger*	Fungus
		*Cryptococcus neoformans*	Encapsulated yeast
		*Neurospora crassa*	Red bread mold
		*Puccinia graminis*	Stem rust
		*Rhizopus microsporus*	Plant pathogen
		*Schizosaccharomyces pombe*	Fission yeast
		*Schizophyllum commune*	Fungus

Note: Vert.: Vertebrates; Inv.: Invertebrates; Dico.: Dicots; Mono.: Monocots.

The first full transcript set (Full Strand-Specific) included all 2000 transcripts as downloaded from the database. For the second, full transcript set (Full Strand-Blind), intended to measure the performance of the predictors in sequences with unknown translation direction, we randomly selected half of the sequences in the previous datasets (1000 transcripts) and replaced it for its reverse complement.

To compare the prediction of complete CDSs, we followed the approach of Tang and collaborators [34], using a set of full transcripts with their respective start codons validated and annotated by Ribo-seq experiments [18]. We, however, extended the number of species in the validation including *Homo sapiens*, *Mus musculus*, *Danio rerio*, *Drosophila melanogaster* and *Arabidopsis thaliana* [21]. For the data previously analyzed by [18], we selected transcripts where the annotated start codon at RefSeq matched to the start codon confirmed by the Ribo-seq data resulting in 5727 and 2701 sequences for *H. sapiens* and *M. musculus*, respectively. On the data analyzed by [21], we considered only the full transcripts with the curated annotation by the Ribo-seq data, which led to 14 193, 20 326, 13 954, 13 653 and 6947 sequences for *H. sapiens*, *M. musculus*, *D. rerio*, *D. melanogaster* and *A. thaliana*, respectively.

For the partial transcripts datasets, we considered that the *de novo* assemblies can result in partial transcripts with no start codon and/or no stop codons. For the first partial transcript set (No Start), we randomly selected, for each transcript of the full transcript dataset, a truncation point in the CDS region and pruned the 5$^\prime $ part, eliminating the start codon. In the second partial transcript set (No Stop), we randomly selected, for each transcript, a new truncation point in the CDS region and pruned the 3$^\prime $ part, eliminating the stop codon. In the third partial transcript set (No Start & No Stop), we randomly selected, for each transcript two truncation points and eliminated the 5$^\prime $ and the 3$^\prime $ ends of the transcript, retaining only part of the CDS region. Cutting points were selected to guarantee a minimum size of 150 nt for the resulting sequences. In cases where the whole transcript was smaller than 150 nt we only pruned the sequence at the start and/or stop codon, depending on the dataset.

We used two different datasets to evaluate Specificity: 3$^\prime $UTR sequences and ncRNA sequences. The 3$^\prime $UTR dataset consisted of the complete 3$^\prime $UTR regions of each transcript in the original full transcript dataset. The 3$^\prime $UTR set was designed to be a real negative set for protein-coding transcriptome projects when the experimental design leads to a selection of mRNA transcripts based on poly-A selection. Additionally, to test the specificity in RNASeq projects with no poly-a specificity, we used ncRNA sequences. For this, we created a dataset containing all ncRNA sequences longer than 200 nt length available for each species in the RFAM database (release 14.1;https://rfam.xfam.org/). To make the specificity test more close to a real transcriptome assembly and fair for the self-training algorithms, we used a mix of sequences containing a proportion of 500 sequences of full-length transcripts and 500 sequences of partial transcripts within the ncRNA sequences.

Moreover, to test CodAn as a classifier of sequences with coding potential, we designed a test by combining all testing sets (i.e. full transcript set, partial transcript set, UTR sequences and ncRNA sequences). We compared the classification performance of CodAn against PLEK (v1.2; [19]), CPAT (v2.0; [37]) and CPPRED (v1.0; [36]) using the standard Precision, Recall and F1-Score values as mentioned above. For this, we considered that TruePositives are coding sequences correctly identified as coding sequences, TrueNegatives are non-coding sequences correctly identified as non-coding sequences, FalseNegatives are coding sequences incorrectly identified as non-coding sequences and FalsePositives are non-coding sequences identified as coding sequences.

## 3 RESULTS AND DISCUSSION

CodAn is a stand-alone software that can be used to reliably predict the location of UTR and CDS regions in full or partial transcripts. CodAn uses two GHMMs, one for a full CDS and another for partial transcripts. GHMMs are used by the most successful gene predictors in use today [2, 16, 32] due to the possibility of accurately modeling each region of the gene using specific probabilistic models and by modeling more accurately the length of each of these regions. With the use of ToPS [14] we could experiment with different model configurations in order to maximize performance. The final model incorporated the best performing of the most successful probabilistic techniques used in genomic gene prediction for the four eukaryotic organism groups. Details on the probabilistic models used for the architecture can be found in Supplemental_Methods.

We compared CodAn’s performance against that of ESTScan [23], TransDecoder [7], Prodigal [11] and GeneMarkS-T [34] in $34$ different organisms of four groups: vertebrates, invertebrates, plants and fungi (Table 1). For each organism, the performance was measured in transcripts of eight different test sets: two sets of strand-specific full transcripts, one set of strand-blind full transcripts and three sets of partial transcripts (‘No Start’, ‘No Stop’ and ‘No Start & No Stop’ and two distinct negative sets, ‘3$^\prime $UTR partial transcripts’ and ‘ncRNA transcripts’).

### 3.1 Prediction accuracy assessment: full transcripts

**Figure 2:**
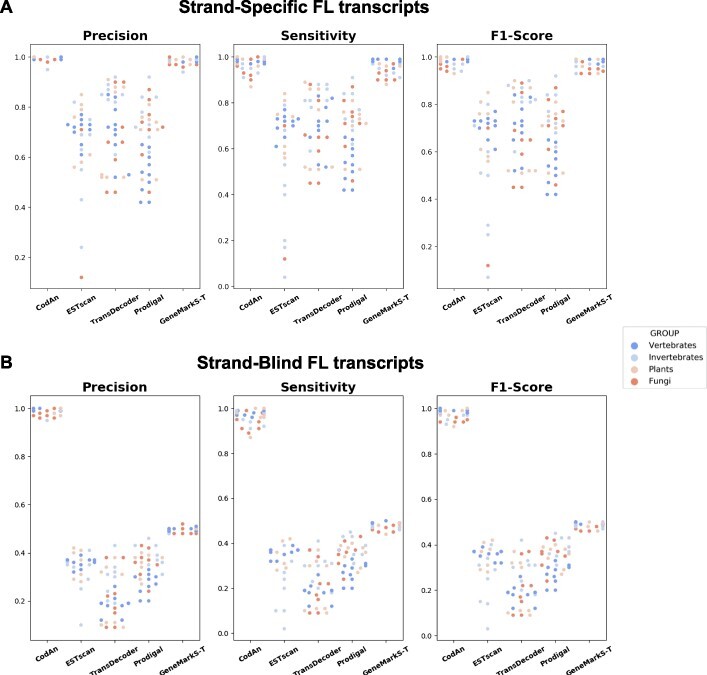
Scatter plot of the full-length (FL) transcript test results. (A) Precision, Sensibility and F1-Score obtained by each tool in the strand-specific FL test. (B) Precision, Sensibility and F1-Score obtained by each tool in the strand-blind FL test. Each dot represents a different organism, color-coded by organism group. Results are grouped vertically by predictor.

For strand-specific stop codon prediction, CodAn presents a higher performance in all four groups as we can see in Table 2. Running F1-scores for each category were all above $97\%$, constantly higher than other approaches. Low standard deviation values in all four organism groups (equal to or lower than 0.01) indicate the robustness of the method. This performance is confirmed when examining a summary of the results for each species, as depicted in Figure 2A, which shows the values obtained for Precision, Sensitivity and F1-score. For complete strand-specific sets, CodAn presented a higher performance for the majority of the organisms in all four categories (Supplemental Table S2_A and Supplemental Table S2_B).

**Table 2:** Average and standard deviation of precision, Recall and F1-score obtained by each tool in the strand-specific full transcript sets analyzed. Bold font highlight the higher value for each group. No results are available for ESTScan in fungal transcripts due to the absence of a fungal model

**Group**	**Predictor**	**Precision**	**Recall**	**F1-score**
Vertebrates	CodAn	**1.00 $\pm $ 0.00**	**0.98 $\pm $ 0.01**	**0.99 $\pm $ 0.01**
	ESTscan	0.71 $\pm $ 0.05	0.71 $\pm $ 0.05	0.71 $\pm $ 0.05
	TransDecoder	0.70 $\pm $ 0.11	0.69 $\pm $ 0.11	0.70 $\pm $ 0.11
	Prodigal	0.53 $\pm $ 0.08	0.53 $\pm $ 0.08	0.53 $\pm $ 0.08
	GeneMarkS-T	0.99 $\pm $ 0.00	**0.98 $\pm $ 0.01**	0.98 $\pm $ 0.01
Invertebrates	CodAn	**0.99 $\pm $ 0.01**	**0.96 $\pm $ 0.03**	**0.97 $\pm $ 0.02**
	ESTscan	0.60 $\pm $ 0.19	0.41 $\pm $ 0.26	0.47 $\pm $ 0.25
	TransDecoder	0.83 $\pm $ 0.09	0.80 $\pm $ 0.09	0.82 $\pm $ 0.09
	Prodigal	0.77 $\pm $ 0.10	0.76 $\pm $ 0.10	0.77 $\pm $ 0.10
	GeneMarkS-T	**0.99 $\pm $ 0.02**	0.95 $\pm $ 0.03	**0.97 $\pm $ 0.02**
Plants	CodAn	**1.00 $\pm $ 0.00**	**0.97 $\pm $ 0.04**	**0.98 $\pm $ 0.02**
	ESTscan	0.71 $\pm $ 0.12	0.70 $\pm $ 0.12	0.70 $\pm $ 0.12
	TransDecoder	0.70 $\pm $ 0.19	0.68 $\pm $ 0.18	0.69 $\pm $ 0.18
	Prodigal	0.69 $\pm $ 0.11	0.69 $\pm $ 0.11	0.69 $\pm $ 0.11
	GeneMarkS-T	0.98 $\pm $ 0.01	0.96 $\pm $ 0.03	0.97 $\pm $ 0.02
Fungi	CodAn	**0.99 $\pm $ 0.01**	**0.95 $\pm $ 0.04**	**0.97 $\pm $ 0.02**
	ESTscan	NA	NA	NA
	TransDecoder	0.67 $\pm $ 0.17	0.64 $\pm $ 0.15	0.65 $\pm $ 0.16
	Prodigal	0.71 $\pm $ 0.13	0.71 $\pm $ 0.13	0.71 $\pm $ 0.13
	GeneMarkS-T	0.98 $\pm $ 0.01	0.94 $\pm $ 0.04	0.96 $\pm $ 0.02

When considering strand-blind sets CodAn significantly outperforms all other applications in stop codon prediction (Table 3; Figure 2B), the F1-scores are at least $40\%$ higher than other approaches, with consistently higher Precision and Recall values in all organisms (Supplemental Table S2_C and Supplemental Table S2_D). In fact, CodAn is the only software for which strand-specific and strand-blind results are almost the same, with F1-score values consistently over $95\%$. Considering that most RNA-seq projects perform sequencing with an unknown orientation of the transcript being sequenced, it is relevant to use predictors that present high precision independently of the orientation of the CDS in the transcripts. We can only speculate on the reasons for this increase in performance, as strand-blind predictions strategies are not always clearly described. In our case we have tried two approaches, one with a single model for predicting CDSs in both strands and one where we used a strand-specific model in the input strand and in its reverse complement, selecting the performance with the highest score. In our case, this last approach had a much better performance.

**Table 3:** Average and standard deviation of precision, Recall and F1-score obtained by each tool in the Strand-Blind full transcript sets analyzed. Bold font highlight the higher value for each group.No results are available for ESTScan in fungal transcripts due to the absence of a fungal model

**Group**	**Predictor**	**Precision**	**Recall**	**F1-score**
Vertebrates	CodAn	**0.99 $\pm $ 0.00**	**0.98 $\pm $ 0.01**	**0.99 $\pm $ 0.01**
	ESTscan	0.36 $\pm $ 0.02	0.36 $\pm $ 0.02	0.88 $\pm $ 0.13
	TransDecoder	0.18 $\pm $ 0.04	0.18 $\pm $ 0.04	0.93 $\pm $ 0.05
	Prodigal	0.27 $\pm $ 0.05	0.27 $\pm $ 0.05	0.93 $\pm $ 0.02
	GeneMarkS-T	0.50 $\pm $ 0.00	0.49 $\pm $ 0.00	0.49 $\pm $ 0.00
Invertebrates	CodAn	**0.99 $\pm $ 0.02**	**0.95 $\pm $ 0.03**	**0.97 $\pm $ 0.02**
	ESTscan	0.31 $\pm $ 0.10	0.21 $\pm $ 0.13	0.24 $\pm $ 0.12
	TransDecoder	0.29 $\pm $ 0.08	0.28 $\pm $ 0.07	0.29 $\pm $ 0.08
	Prodigal	0.39 $\pm $ 0.05	0.39 $\pm $ 0.05	0.39 $\pm $ 0.05
	GeneMarkS-T	0.49 $\pm $ 0.01	0.48 $\pm $ 0.01	0.48 $\pm $ 0.01
Plants	CodAn	**0.99 $\pm $ 0.01**	**0.96 $\pm $ 0.04**	**0.98 $\pm $ 0.02**
	ESTscan	0.35 $\pm $ 0.06	0.35 $\pm $ 0.06	0.35 $\pm $ 0.06
	TransDecoder	0.22 $\pm $ 0.13	0.21 $\pm $ 0.12	0.21 $\pm $ 0.12
	Prodigal	0.35 $\pm $ 0.05	0.35 $\pm $ 0.05	0.35 $\pm $ 0.05
	GeneMarkS-T	0.49 $\pm $ 0.01	0.48 $\pm $ 0.02	0.49 $\pm $ 0.01
Fungi	CodAn	**0.98 $\pm $ 0.01**	**0.94 $\pm $ 0.04**	**0.96 $\pm $ 0.02**
	ESTscan	NA	NA	NA
	TransDecoder	0.21 $\pm $ 0.11	0.20 $\pm $ 0.10	0.21 $\pm $ 0.11
	Prodigal	0.36 $\pm $ 0.06	0.35 $\pm $ 0.06	0.36 $\pm $ 0.06
	GeneMarkS-T	0.49 $\pm $ 0.01	0.47 $\pm $ 0.02	0.48 $\pm $ 0.01

### 3.2 Prediction accuracy assessment: experimentally validated strand-specific full transcripts

Next, we evaluated the accuracy for complete CDS prediction using a set of full transcripts of *H. sapiens*, *M. musculus*, *D. rerio*, *D. melanogaster* and *A. thaliana* with their respective start codons validated and annotated by Ribo-seq experiments [18, 21].

**Figure 3:**
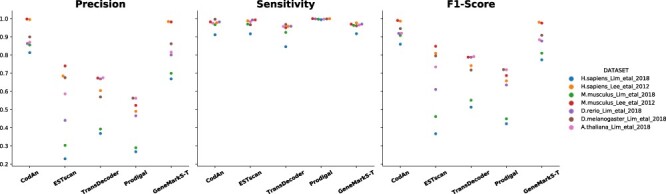
Scatter plot of the precision, sensitivity and F1-score obtained by each tool in the Ribo-seq experimentally validated datasets considering the full CDS region. Each dot represents a different dataset, color-coded by species dataset. Results are grouped vertically by predictor.

The tests revealed that GeneMarkS-T and CodAn presented higher performance than the other tools, but this time with a clear advantage for CodAn, with a higher percentage of correct predictions in five of the seven datasets and small advantage in two (Table 4, Figure 3; Supplemental Table S2_E). CodAn presented higher rates of correct predictions and an almost perfect score for predicting the stop codon position (over $97\%$ of the predictions in all datasets). These results confirm the consistent advantage of CodAn in full CDS prediction obtained in the first 34 datasets (Supplemental Table S2_A and Supplemental Table S2_B). There is a significant increase in correct CDS predictions with CodAn, when compared to the next best performing software, GeneMarkS-T: CodAn presented an average of 235 more correct CDS predictions out of the 2000 genes in the dataset, averaging a 17$\%$ increase (Table S2_A at supplemental_Table_S2)

**Table 4:** Precision, recall and F1-score for the prediction in datasets with start codons confirmed by ribo-seq experiments. True positives are sequences with the whole CDS predicted correctly (start and stop codon). Bold font highlight the higher value for each dataset. The ‘Size’ column refers to the number of transcripts in the dataset

**Dataset**	**Size**	**Predictor**	**Precision**	**Recall**	**F1-score**
*H.sapiens*	14193	CodAn	**0.81**	**0.75**	**0.78**
(Lim *et al.*, 2018)		ESTScan	0.23	0.22	0.23
		TransDecoder	0.37	0.35	0.36
		Prodigal	0.27	0.27	0.27
		GeneMarkS-T	0.67	0.63	0.65
*H.sapiens*	5727	CodAn	**0.99**	**0.97**	**0.98**
(Lee *et al.*, 2012)		ESTScan	0.68	0.68	0.68
		TransDecoder	0.60	0.59	0.60
		Prodigal	0.49	0.49	0.49
		GeneMarkS-T	0.98	0.96	0.97
*M.musculus*	20326	CodAn	**0.85**	**0.83**	**0.84**
(Lim *et al.*, 2018)		ESTScan	0.30	0.30	0.30
		TransDecoder	0.39	0.38	0.39
		Prodigal	0.29	0.29	0.29
		GeneMarkS-T	0.70	0.68	0.69
*M.musculus*	2701	CodAn	**1.00**	**0.98**	**0.99**
(Lee *et al.*, 2012)		ESTScan	0.74	0.74	0.74
		TransDecoder	0.67	0.65	0.66
		Prodigal	0.52	0.52	0.52
		GeneMarkS-T	0.97	0.95	0.97
*D.rerio*	13954	CodAn	**0.86**	**0.85**	**0.86**
(Lim *et al.*, 2018)		ESTScan	0.44	0.44	0.44
		TransDecoder	0.67	0.65	0.66
		Prodigal	0.47	0.47	0.47
		GeneMarkS-T	0.80	0.78	0.79
*D.melanogaster*	13653	CodAn	**0.90**	**0.90**	**0.90**
(Lim *et al.*, 2018)		ESTScan	0.68	0.66	0.67
		TransDecoder	0.57	0.56	0.56
		Prodigal	0.56	0.56	0.56
		GeneMarkS-T	0.86	0.83	0.85
*A.thaliana*	6947	CodAn	**0.87**	**0.85**	**0.86**
(Lim *et al.*, 2018)		ESTScan	0.59	0.58	0.58
		TransDecoder	0.67	0.65	0.66
		Prodigal	0.56	0.56	0.56
		GeneMarkS-T	0.82	0.79	0.80

**Figure 4:**
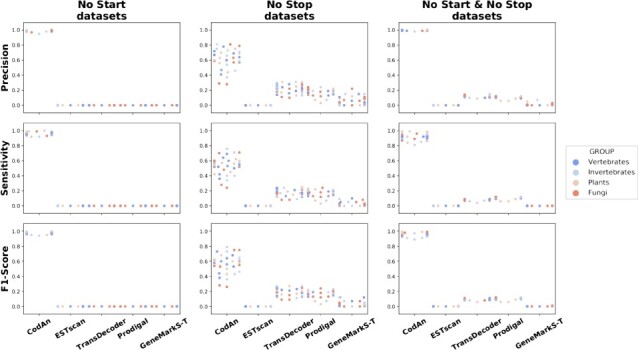
Scatter plot of the partial transcript test results. The plots are showing the Precision, Sensitivity and F1-Score obtained by each tool on ‘No Start’, ‘No Stop’ and ‘No Start & No Stop’ tests performed on all species analyzed in the present study. Each dot represents a different organism, color-coded by organism group.

**Figure 5:**
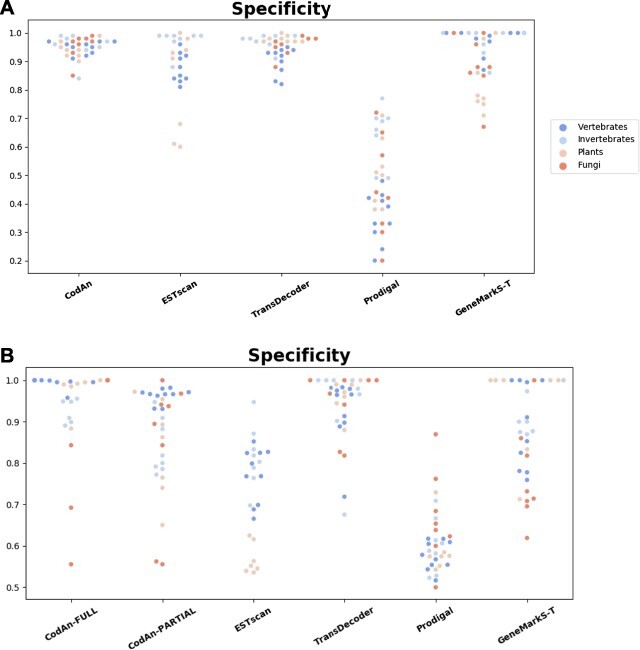
Scatter plot of Specificity obtained by each tool in the prediction. We used two negative datasets (A) 3$^\prime $UTR region datasets (using only the partial model of CodAn) and (B) ncRNA datasets (using the full and partial models of CodAn). Each dot represents a different organism, color-coded by organism group. Results are grouped vertically by predictor.

In summary, for full transcripts, CodAn significantly outperforms other available software in predicting CDS for the full transcript of unknown orientation and increases precision in full CDS prediction, while still matching the best stop codon precision measurements. This indicates that CodAn it the best choice when the annotation of the whole coding sequence is necessary for the subsequent analysis.

### 3.3 Prediction accuracy assessment: partial transcripts

In most real-life situations, transcriptome sequencing projects performing *de novo* assemblies produce a high rate of partial transcripts [9]. It is therefore relevant to measure the accuracy of predictions also for these sequences. To form a more precise picture we separately measure the accuracy for prediction in transcripts consisting of: (i) only CDS nucleotides; (ii) 5$^\prime $UTR and CDS nucleotides; and (iii) CDS and 3$^\prime $UTR nucleotides. These datasets presented a much harder challenge for all of the applications used in the comparison (Figure 4; Supplemental Table S2_F). For the NoStart dataset, only CodAn was able to correctly identify a significant number of CDSs, in this case with very good results, averaging over $97\%$ in F1 score values. The situation changed for the NoStop datasets but still with a clear advantage for CodAn: average F1 score for CodAn was above $58\%$, in comparison to a maximum of $27\%$ for the other applications. Finally, for the NoStartNoStop (CDS only) sequences, CodAn F1 scores were, again, averaged more than $96\%$, while F1 scores for the other applications were always below $13\%$.

These results showed that other approaches fail to obtain even modest precision or recall rates for the prediction of CDSs in partial transcripts, with CodAn achieving consistently higher rates. This clearly indicates CodAn as the best approach to handle cases where the partial transcripts are highly abundant in the transcriptome assembly. In fact, it is a common feature in *de novo* assemblies in which partial transcripts can represent up to $50\%$ of the sequences assembled [9].

**Figure 6:**
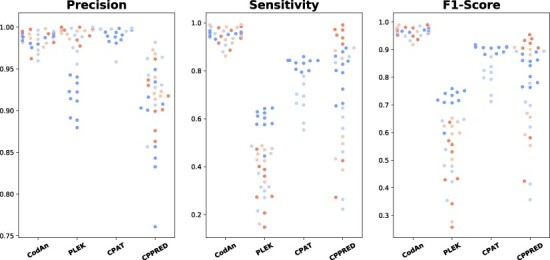
Scatter plot for Precision, Specificity and F1-score obtained by CodAn, PLEK, CPAT and CPPRED. Each dot represents a different organism, color-coded by organism group, whereas blue, light blue, light orange and orange represent vertebrates, invertebrates, plants and fungi, respectively. Results are grouped vertically by predictor.

### 3.4 False-positive assessment using partial 3ʹUTR and ncRNA transcripts

Different sequencing protocols can produce two types of negative sequences when considering CDS prediction: UTR-only sequences or ncRNA sequences.

To estimate the rate in which such transcripts have false-positive predictions in the first case we ran all applications in the 3$^\prime $UTR sequences of the previous datasets. The results show that CodAn and TransDecoder, as a rule, presented the lowest number of false-positives, whereas Prodigal presented the highest number of false-positives (Figure 5A; Supplemental Table S2_G). TransDecoder presented the best overall performance with better average specificity values for invertebrates ($95\%$ versus $90\%$), plants ($95\%$ versus $90\%$) and fungi ($95\%$ versus $90\%$). The only exception was for vertebrates with GeneMarkS-T presenting a Specificity of $97\%$, against $95\%$ of CodAn and $90\%$ of TransDecoder.

Specificity assessment for ncRNA sequences showed that the full transcript model of CodAn presents the best performance of all predictors. If instead, we use the partial model of CodAn, its performance is higher for vertebrates, whereas Transdecoder presented a slightly better performance in the other groups (Figure 5B; Supplemental Table S2_H). Overall, both models of CodAn presented satisfactory results on specificity tests.

### 3.5 CodAn as a coding potential classifier

The high performance obtained by CodAn on the specificity tests of the previous section indicates that CodAn can be also potentially used for evaluating the coding potential of sequences and can be used to classify sequences as coding or non-coding. This is a slightly different problem than identifying the coding region of a transcript, but finding a full or partial CDS in a sequence can lead to classify it as coding or non-coding. The coding potential tools developed are classifiers, which only indicate if a sequence has the potential of being coding or non-coding. In this sense, we compared CodAn with three different coding potential classifiers using all datasets designed in the testing set (i.e. full-length transcripts, partial transcripts, UTR transcripts and ncRNA transcripts). The results revealed that CodAn outperformed the competition, associating high precision values with clearly superior sensitivity (Figure 6; Supplemental Table S2_I). These data indicate that CodAn is suitable for the coding potential classification task.

### 3.6 Running time

We measured the processing times of CodAn, ESTscan, TransDecoder, Prodigal and GeneMarkS-T by running the predictions on a Full-Length transcript dataset containing 2000 sequences and using a personal computer (Intel 6-Core i7 with 16-Gb memory). The test revealed that CodAn processed the set of sequences in 32 s (i.e. about 0.016 s per sequence) when using a single CPU. Despite being slower than its competitors (i.e. 16, 9, 6 and 5 s for Prodigal, Transcoder, ESTScan and GenenmarkS-T, respectively), CodAn is still fast enough to process large datasets in personal computers. For example, 200 000 sequences will be processed in about 53 min by using one CPU. Additionally, CodAn has an option to use multiple CPUs that can significantly increase the time performance. For instance, with the use of four CPUs, the 200000 sequences would be processed in only 17 min.

## 4 CONCLUSION

We presented CodAn, a software that generates highly confident transcript characterization for a wide range of eukaryote organisms. Currently, CodAn has four specific prediction models: vertebrates, invertebrates, fungi and plants. CodAn was tested in a variety of situations for transcript annotations in 34 different organisms: full strand-specific stop codon characterization, full strand-specific CDS characterization, full strand-blind and partial sequences that excluded either the start codon, the stop codon or both. In all but the first measure, CodAn obtained a clear advantage over other software, in particular on partial and strand blind sequences. Even for the prediction of stop codons in strand-specific full sequences, CodAn matched or had a slight advantage over the current gold-standard predictor, GeneMarkS-T. This high confidence is achieved by the use of multiple probabilistic models integrated using a GHMM. The design of CodAn was based on the development of model parameters for four groups of eukaryotes: vertebrates, invertebrates, plants and fungi. Each parameter set was estimated based on a mix of reference transcripts from several species of one of the organism groups. CodAn can run on any desktops/laptops or take advantage of large multi-processor servers based on UNIX OS.

We showed that these generic models work well and result in a reliable characterization of transcripts in a wide range of eukaryote species. Considering the datasets used in the present analysis, CodAn had a clear performance advantage when considering all common situations of RNA sequencing projects, in particular with strand-blind full sequences and partial sequences. Also, even in strand-specific prediction where CodAn and GeneMarkS-T presented similar stop-codon prediction performance, CodAn presented a significant increase in fully-correct CDS.

In summary, our data indicate that CodAn is the best approach to be applied on studies focusing to characterize the CDS regions and the UTR landscape of partial and/or full transcripts and can help the improvement of current and future gene annotation for transcriptomes of eukaryote species, which is a field under constant expansion [28].

##

**5 KEY POINTS**

- CodAn is a CDS prediction software that performs confident transcript characterization.
- A comprehensive analysis using data from 34 organisms revealed that CodAn is suitable for use on a wide range of eukaryote species, including plants, fungi, vertebrates and invertebrates such as insects, *C. elegans*, anemone, *S. mansoni* and *C. intestinalis*.
- CodAn improved the accuracy of whole CDS prediction in transcripts with a known or unknown strand.
- CodAn improved the accuracy of CDS prediction in partial sequences.
- CodAn presents higher performance than the competition on any scenario of transcriptome assembly.

## Supplementary Material

Supplemental_Table_S1_1_bbaa045

Supplemental_Table_S2_bbaa045

Supplemental_Figure_1_bbaa045

Supplemental_Figure_2_bbaa045

Supplemental_Methods_1_bbaa045

## Notes

**Pedro G. Nachtigall** received his PhD in Genetics from the UNESP (Universidade Estadual Paulista, Brazil) in the year 2017. Currently, he is a postdoctoral researcher at Instituto Butantan, São Paulo, Brazil. His research focus is in Comparative Genomics, Transcriptome and Evolutionary Biology.

## Notes

**André Y. Kashiwabara** received his PhD in Computer Science from the Universidade de São Paulo and is currently and Adjunct Professor at the Department of Computer Science at the Universidade Tecnologica Federal do Paraná (UTFPR), Brazil. His research focus on development of machine learning approaches for genomics and transcriptomics.

## Notes

**Alan M. Durham** received his PhD in Computer Science from the University of Illinois at Urbana-Champaign and is currently an associate professor of the Universidade de São Paulo, Brazil. His research focus in the development of probabilistic object-oriented frameworks and their application in genomics and transcriptomics bioinformatics software.
