# Supplementary material for: CodAn: predictive models for precise identification of coding regions in eukaryotic transcripts
Source: Brief Bioinform. 2020 May 27;22(3):bbaa045. doi: 10.1093/bib/bbaa045 (PMC8138839; doi:10.1093/bib/bbaa045)
Supplement: Supplemental_Figure_1_bbaa045 [file supplemental_figure_1_bbaa045.pdf]

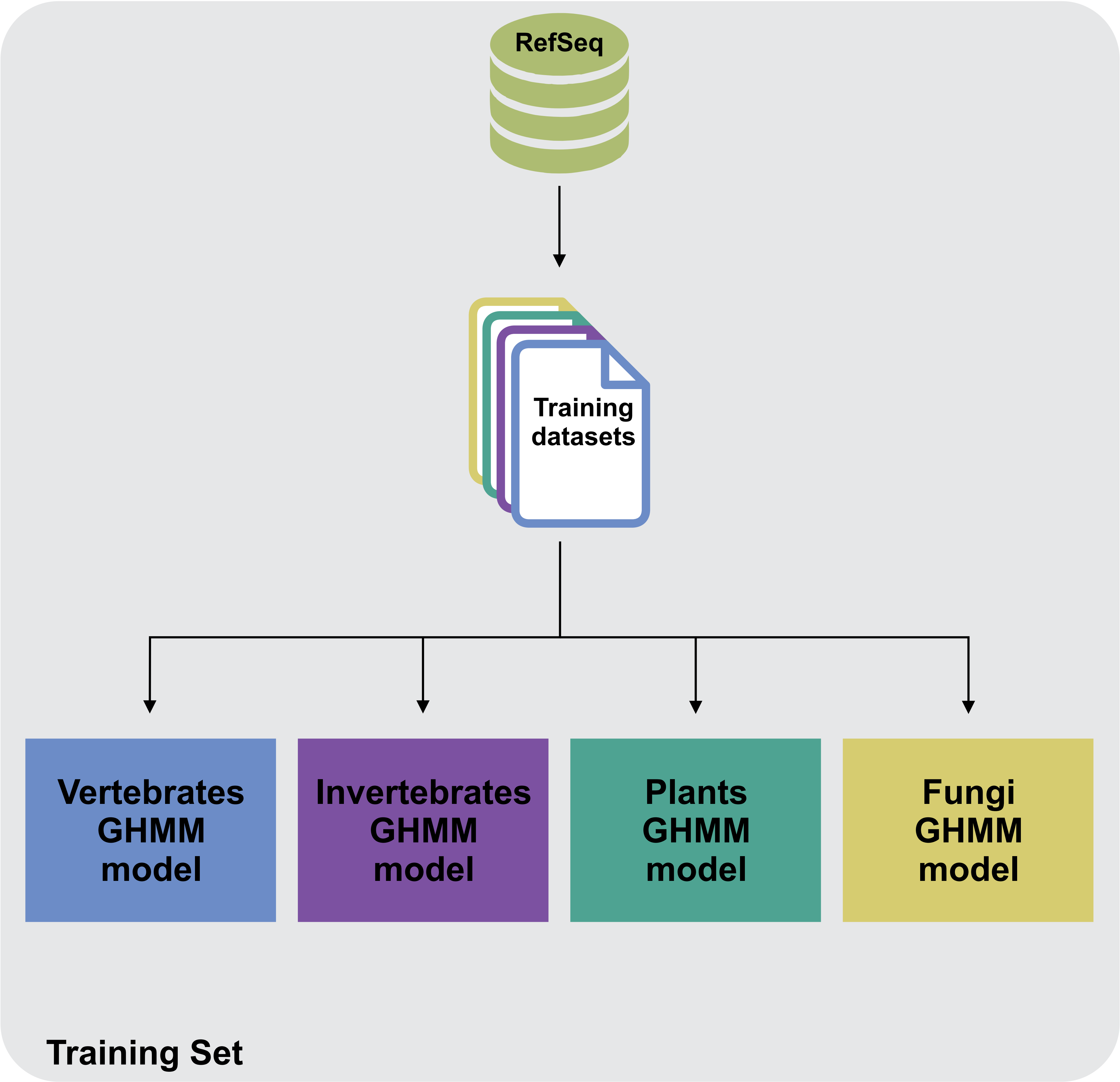
RefSeq
Training datasets
Vertebrates GHMM model
Invertebrates GHMM model
Plants GHMM model
Fungi GHMM model
Training Set
