# Supplementary material for: CodAn: predictive models for precise identification of coding regions in eukaryotic transcripts
Source: Brief Bioinform. 2020 May 27;22(3):bbaa045. doi: 10.1093/bib/bbaa045 (PMC8138839; doi:10.1093/bib/bbaa045)
Supplement: Supplemental_Figure_2_bbaa045 [file supplemental_figure_2_bbaa045.pdf]

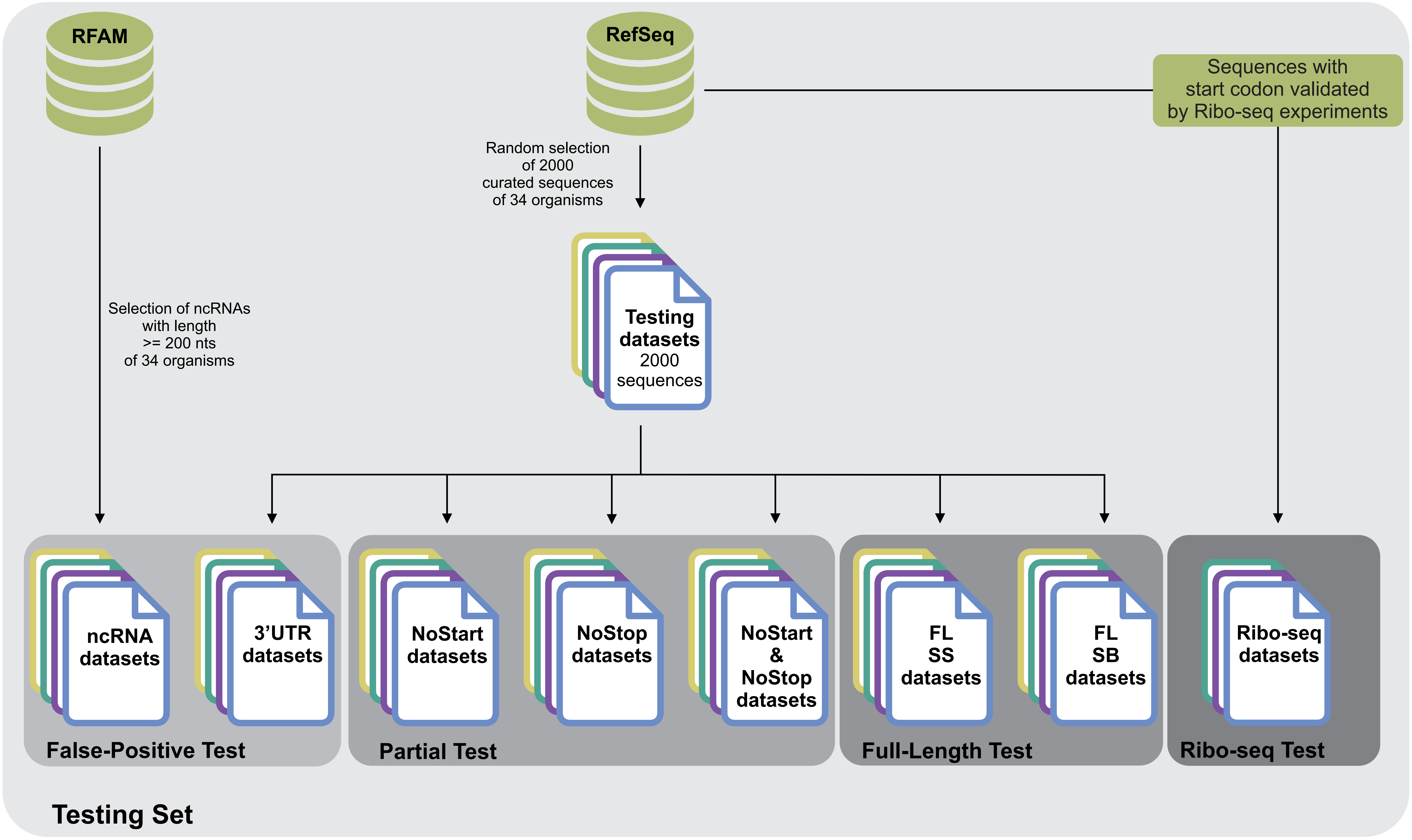
RFAM
RefSeq
Sequences with start codon validated by Ribo-seq experiments
Random selection of 2000 curated sequences of 34 organisms
Selection of ncRNAs with length >= 200 nts of 34 organisms
Testing datasets 2000 sequences
ncRNA datasets
3'UTR datasets
False-Positive Test
NoStart datasets
NoStop datasets
NoStart & NoStop datasets
Partial Test
FL SS datasets
FL SB datasets
Full-Length Test
Ribo-seq datasets
Ribo-seq Test
Testing Set
